# Measuring the blockade of malaria transmission: analyzing the results of mosquito feeding assays

**DOI:** 10.1186/1475-2875-11-S1-P18

**Published:** 2012-10-15

**Authors:** Thomas S Churcher, Andrew M Blagborough, Robert E Sinden

**Affiliations:** 1 Department of Infectious Disease Epidemiology, Imperial College London, London W2 1PG, UK; 2 Department of Life Sciences, Imperial College London, South Kensington, London SW7 2AZ, UK; 3 The Jenner Institute, University of Oxford, Roosevelt Drive, Oxford, OX3 7DQ, UK

## Background

Mosquito Feeding Assays are currently the only method used to assess the effectiveness of malaria transmission blocking interventions (TBIs) currently under development. Infectious gametocytes are fed to mosquitoes which are then dissected after a fixed time interval to determine how efficiently oocysts have developed on the midgut. Feeding assays include The Standard Membrane Feeding Assay (SMFA), the Direct Membrane Feeding Assay and the Direct Feeding Assay, which use different methods of parasite presentation but all assess mosquito infectivity in the same way. Operation and analysis of these assays varies between laboratories: field scientists often measure TBI efficacy as reduction in the prevalence of infected mosquitoes whilst laboratory scientists are more likely to quote efficacy as a change in the number of oocysts within the mosquito. These metrics give outputs that differ widely, resulting in need for greater understanding of how these feeding assay SMFA inform TBI assessment.

## Materials and methods

Data from 538 different SMFAs (conducted on *Plasmodium falciparum* and *P. berghei*, in either *Anopheles gambiae* or *A. stephensi*) is used to illustrate why generalized linear mixed models should be used to analyze mosquito feeding assays data.

## Results

The relationship between oocyst prevalence and intensity is complex, yet predictable. We demonstrate that the distribution of oocysts between mosquitoes is highly over-dispersed, making efficacy estimates based on reductions in intensity highly uncertain. Analysis of 30 feeding assays carried out on the same TBI confirms that the observed reduction in prevalence depends upon the parasite exposure (as measured by oocyst intensity in the control group), with assays which have lower exposure appearing more effective. By contrast, if efficacy is estimated as a reduction in oocyst intensity, then this candidate demonstrated constant efficacy, irrespective of the exposure level.

## Conclusions

To report transmission-blockade efficacy accurately, the results of membrane feeding assays should give both the prevalence and intensity of oocysts in both the control and intervention group. Candidates should be assessed against a range of parasite exposures to allow laboratory results to be extrapolated to different field situations. Currently, many studies assessing TBIs are underpowered and uncertainties in efficacy estimates rarely reported. Statistical techniques that account for oocyst over-dispersion can reduce the number of mosquitoes that need to be dissected and allow TBI candidates from different laboratories to be accurately compared.

